# Disrupted Resting-State Functional Connectivity between the Dorsal Attention, Default Mode, and Frontoparietal Networks in Nonorganic Gastrointestinal Disorder Patients with Spleen Deficiency Syndrome

**DOI:** 10.1155/2021/6681903

**Published:** 2021-05-07

**Authors:** Yanzhe Ning, Wenbin Jia, Dongqing Yin, Xinzi Liu, Hong Zhu, Hongxiao Jia

**Affiliations:** ^1^The National Clinical Research Center for Mental Disorders & Beijing Key Laboratory of Mental Disorders, Beijing Anding Hospital, Capital Medical University, Beijing, China; ^2^Advanced Innovation Center for Human Brain Protection, Capital Medical University, Beijing, China; ^3^Department of Preschool Education, School of Education, Hangzhou Normal University, Hangzhou, China

## Abstract

**Introduction:**

Spleen deficiency syndrome (SDS), a common clinical syndrome of traditional Chinese medicine, is manifested with digestive symptoms and cognitive impairments. However, the cognitive neural mechanism in brain networks of SDS still remained unclear. Our aim was to investigate the changes between the default mode, dorsal attention, and frontoparietal networks in SDS.

**Methods:**

Twenty nonorganic gastrointestinal disorder (NOGD) patients with SDS and eighteen healthy controls were enrolled to attend functional magnetic resonance imaging scan and participated a continuous performance test (CPT) before scanning.

**Results:**

Compared with healthy controls, NOGD patients with SDS showed the significantly increased functional connectivity (FC) between dorsal attention network (DAN) and left frontal-parietal control network (LFPN) and significantly decreased FC between LFPN and default mode network (DMN). The functional network connectivity analysis showed positive correlation coefficients between the DAN and LFPN and DAN and DMN as well as negative correlation between LFPN and DMN in NOGD patients with SDS compared with healthy controls. Correlation analysis revealed that the increased FC between LFPN and DAN was positively correlated with 4-digitnumber reaction time mean (RTM) and 3-digitnumber RTM.

**Conclusion:**

Our study may provide novel insights into the relationship among the DMN, DAN, and FPN in NOGD patients with SDS to deepen our understanding of the neuropsychological mechanisms of SDS.

## 1. Introduction

Spleen deficiency syndrome (SDS) is a common clinical syndrome of traditional Chinese medicine (TCM) in the digestive system diseases, which cannot only manifest with the edema and diarrhea but also cognitive impairments according to the Huangdi Neijing [1]. In the recent years, proteomic, metabonomic, and genomic technologies have been applied to study intrinsic mechanisms of SDS [2]. One study exploit high-throughput miRNA sequencing to detect pathogenesis of chronic superficial gastritis patients with SDS and detected eleven candidate serum miRNAs [3]. Moreover, another study compared differences of proteins between SDS and damp-heat syndrome at the level of proteomics [4]. However, neuropsychological studies on SDS are poorly interpreted.

Nonorganic gastrointestinal disorders (NOGDs) are worldwide epidemic digestive system diseases [5]. The main symptoms are gastrointestinal discomfort, such as vomiting, diarrhea, constipation, and abdominal distension, which meet the clinical manifestation of SDS. NOGDs are a series of functional disorders, mainly including functional dyspepsia, irritable bowel syndrome, and chronic nonatrophic gastritis, which could eliminate the influence of organic impairments on SDS [6]. Moreover, compared with healthy subjects with SDS, NOGDs with SDS were more easily defined and affected by less mixed factors. Hence, NOGDs with SDS are right to study neuropsychological mechanisms of SDS.

The functional magnetic resonance imaging (fMRI), a noninvasive neuroimaging technology, has been extensively applied to explore the neuropsychological mechanisms of different neuropsychiatric diseases and TCM patterns (Zheng) [7–9]. The study on erectile dysfunction patients with two TCM patterns showed different cerebral activities between SDS and kidney-yang deficiency pattern, which may interpret the neuromechanism of TCM patterns [8]. One fMRI study on SDS showed abnormal amplitude of low-frequency fluctuations (ALFF) in the lateral occipital, temporal occipital fusiform and frontal pole cortices, which were also significantly correlated with scores of clinical memory scale [10]. Another study revealed altered FCs of the DMN in subhealthy patients with SDS [11]. However, few studies focused on altered resting networks in SDS. Numerous resting brain networks were detected in the resting state, such as the sensorimotor network, the frontal-parietal control network (FPN), the salience network (SN), the dorsal attention network (DAN), the salience network, and the default mode network (DMN). The DMN is active by intrinsic neuronal activity during the resting state, which is related to attending external and internal stimuli known as the “task-negative” network, whereas the DAN and FPN are both task-related networks, known as “task-positive” networks. These three brain networks are neurocognitive networks. One recent neuroimaging study revealed abnormal functional connectivities within the DMN in functional dyspepsia [12]. However, there are no studies focused on interactions between the DAN, DMN, and FPN in SDS.

In the current study, to detect alteration interactions among the DAN, FPN, and DMN, we employed NOGDs with SDS and healthy controls (HCs). The independent component analysis (ICA) was employed to detect the three resting-state brain networks. Then, we conducted the FC analysis and functional network connectivity (FNC) analysis sequentially to study the interactions among the DMN, DAN, and FPN. Furthermore, the linear regression analysis was conducted to evaluate the correlation between the cognitive assessments and FC coefficients in NOGD patients with SDS. The study may supply evidence for the relationship between enteric nervous system and brain function.

## 2. Materials and Methods

Our study had been approved by the Ethics Committee of Beijing Anding Hospital. All participants had signed informed consent before attending this study.

### 2.1. Participants

Twenty right-handed subjects (aged 25.80 ± 3.41 years) were diagnosed as NOGDs by gastroscopy according to the Consensus on Integrated Traditional Chinese and Western Medicine in the Treatment of Chronic Gastritis and the ROMA IV criteria [6] and diagnosed as SDS based on the1986 deficiency syndrome of criteria of TCM by Integrative Medicine Deficiency Syndrome and Senile Diseases Research Association [13]. Meanwhile, the patients should also meet the following criteria below: aged from 18 to 45 years and the duration of both NOGDs and SDS for more than six months. The exclusion criteria were as follows: diagnosed as gastrointestinal disorders with organic pathologic changes; with history of psychiatric disorders; with history of alcohol or drug abuse; and any MRI contraindication. Another 18 subjects were recruited as healthy controls (aged 27.28 ± 3.61 years) with no history of psychiatric and neurological disorders and no clinical diagnosis of NOGDs and SDS.

### 2.2. MRI Acquisition

A 3.0 Tesla MRI scanner (Trio, Siemens, Germany) was employed to acquire images at the Imaging Center for Brain Research in Beijing Normal University. Twenty patients and 18 healthy controls participated in the MRI scanning. Before scanning, the enrolled subjects were all required to have a rest for 30 minutes and ordered to keep eyes closed, awake and stay still during the entire scanning course. All MRI scans have been conducted between 3 p.m. and 5 p.m.

Prior to the fMRI scanning, structural data for anatomical localization would be detected. The single-shot, gradient-recalled echo-planar imaging sequence was applied with the parameters as follows: repetition time = 2000 ms, echo time = 30 ms, flip angle = 90°, matrix = 64 × 64, slice thickness = 3.5 mm, field of view = 200 mm × 200 mm, gap = 1 mm, 33 axial sections, and 240 volumes. In our study, a first 480-second resting scan and then 250-second high-resolution structural scan were employed.

### 2.3. Continuous Performance Test

To assess the attention, a continuous performance test (CPT) from the Chinese version of MATRICS Consensus Cognitive Battery (MCCB) through the computer was applied in our clinical neuropsychological assessments [14]. We recruited 20 NOGDs with SDS and 18 healthy subjects taking part in the attention function test. The numeric types including 2- to 4-digit numbers (0 to 9) will appear on the computer screen one at a time every 500 ms and a 500-ms interval. The participant left-clicks the mouse as soon as possible when the number is same as the previous number presented in the screen. Responses to these stimuli will be recorded. After the participant finished the test, the results would be stored in the file of computer. The actual number of responses (ANR), false trials (FT), standard deviation (SD), and reaction time mean (RTM) are selected as assessment indicators to appraise the attention.

### 2.4. Data Processing and Analysis

Data Processing Assistant for Resting-State fMRI (DPARSF, http://rfmri.org/DPARSF) was applied during the preprocessing step. We firstly discarded the first 10 volumes for signal equilibrium and corrected 230 volumes for slice timing. Then, we conducted the following steps: spatial realignment for head motions, normalization into the MNI template, resampling into 3 × 3 × 3 mm^3^ voxels, smoothing with a Gaussian kernel of 8 mm full width, and spurious variances (head motion, ventricular and white matter signal, and the derivatives of each of these signals) reduction. Finally, the linear trends were removed from the time courses. In addition, two patients with exhibiting head motion >2°maximum rotation and translation >2 mm were excluded.

After preprocessing, we used the GIFT software package (GIFT v4.0a) [15] to conduct group ICAs. The group ICAs were simultaneously conducted on the NOGD and HC groups to ensure consistency. There were four main steps for this analysis, which included individual components analysis, group principle component analysis, ICA, and back reconstruction. Data were decomposed into 20 components using the minimum description length (MDL) criteria [16]. Then, the four components (LFPN, RFPN, DAN, and DMN) were automatically selected via a template-matching algorithm. Each component was highly correlated and spatially overlapped with the standard templates. At last, the four components were manually inspected by two experts (WB and YZ). The result was further confirmed by visual inspection with a consensus of the experts (WB and YZ).

#### 2.4.1. FC Analysis

In order to get the functional connectivity between the subnetworks (LFPN, RFPN, DAN, DMN) of the NOGD and HC groups, we first performed Fisher's r-to-z transformation on the four components and then calculated the Person correlation between them. Then, the two-sample *t*-test was employed to compare the functional connectivity between subnetworks of the NOGD and HC groups, with age and gender as covariates. The Gaussian random field (two-tailed) method was used to correct for multiple comparisons at the voxel level (*p* < 0.01) and cluster level (*p* < 0.05).

#### 2.4.2. FNC Analysis

We are also interested in the temporal correlations between the components (brain networks) obtained by the ICA and the difference between the NOGD and HC groups [15]. Hence, FNC toolbox (v2.3) was used to find and display the temporal correlations between the four components. The time courses from the four components for all subjects were first interpolated to enable detection of sub-TR hemodynamic delay differences [17]. We examined all 4!/(2!(4 − 2)!) = 6 possible combinations, and the direct possibility was calculated through the constrained maximal lagged correlation coefficient between each pair of subnetworks. Assumed that $\bar{X}$ occurs at the initial reference point *i*_0_ (${\bar{X}}_{i_{0}}$) and that $\bar{Y}$ circularly shifted Δ_*i*_ units from reference point of *i*_0_ (${\bar{Y}}_{i_{0}+\Delta_{i}}$), then individual correlations (*ρ*_Δ_*i*__) of two time courses can be calculated as follows:

(1) $$\begin{array}{c} \rho_{\Delta_{i}}=\frac{\left({\bar{X}}_{i_{0}}^{T}\right)\left({\bar{Y}}_{i_{0}+\Delta_{i}}\right)}{\sqrt{\left({\bar{X}}_{i_{0}}^{T}{\bar{X}}_{i_{0}}\right)}\;\times \;\sqrt{{\bar{Y}}_{i_{0}+\Delta_{i}}^{T}\;{\bar{Y}}_{i_{0}+\Delta_{i}}}}. \end{array}$$

Here, *T* represents the number of time points, and Δ_*i*_ represents the noninteger change in time (lag time, maximal *t* = 2 TR). The lag between time courses${\bar{X}}_{i_{0}}$ and ${\bar{Y}}_{i_{0}+\Delta_{i}}$ is Δ_*i*_ in seconds. Therefore, vectors *ρ* can be calculated between time courses $\bar{X}$ and $\bar{Y}$. The maximal correlation value of all ${\bar{\rho }}_{\Delta_{i}}\;$and corresponding lag, $\delta_{{\bar{\rho }}_{\max}}$, were saved for time courses $\bar{X}$ and $\bar{Y}$ [18].The lag values represent the amount of delay between two correlated time courses (subnetworks) averaged across the NOGD and HC groups. We calculated all 6 pairwise combinations and then performed the one-sample *t*-test to define the significance of the combination (*p* < 0.05, false discovery rate corrected). Two-sample *t*-tests were subsequently applied to detect the abnormal connections within the subnetworks and between the subnetworks, with age and gender as covariates.

### 2.5. Correlation Analysis

To detect the relationship between abnormal FC values and CPT scores, we performed the correlation analysis by a partial correlation analysis, while controlling age, education, and gender. The relationship was significant if *p* < 0.05 (false discovery rate corrected).

## 3. Results

### 3.1. Demographic and Clinical Information

Demographic data of all subjects are displayed in Table 1. NOGD patients with SDS and healthy controls showed no statistical difference in age, gender, and education level (*p* > 0.05). Then, we conducted CPT scores between the two groups (summarized in Table 2). NOGD patients with SDS showed decreased scores of 2-FT, 2-RTM, and 4-RTM compared with HCs.

### 3.2. Spatial Distribution of the DMN, FPN, and DAN

**Figure 1:**
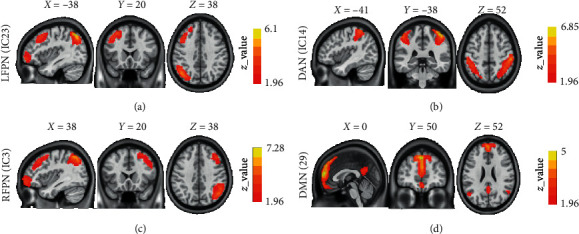
Spatial distribution of the DAN, FPN, and DMN. *Note.* DAN, dorsal attention network; DMN, default mode network; FNC, functional network connectivity; IC, independent component; LFPN, left frontal-parietal control network; RFPN, right frontal-parietal control network.

The functional data were divided into 20 ICs by the group ICA. After spatial correlation with the network template and identification by the experts, IC3, IC14, and IC23 were identified as the RFPN, DAN, and LFPN, respectively (shown in Figure 1). We also found that IC29 was identified as the DMN.

### 3.3. FC Analysis

**Figure 2:**
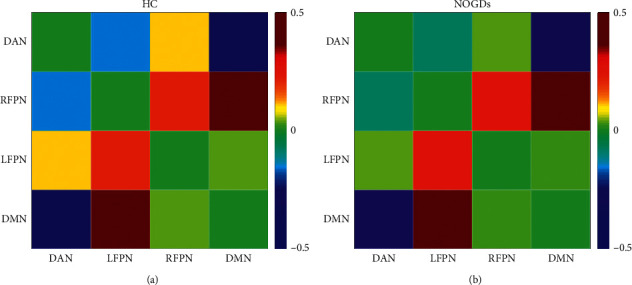
The matrix FC of RFPN, DAN, LFPN, and DMN in NOGDs and HCs. DAN, dorsal attention network; DMN, default mode network; FNC, functional network connectivity; HC, healthy control; LFPN, left frontal-parietal control network; RFPN, right frontal-parietal control network.

During the resting state, the matrix FCs of RFPN, DAN, LFPN, and DMN in NOGDs and HCs (one-sample *t*-test) are shown in Figure 2. The two-sample *t*-test showed that the FC between DAN and LFPN was significantly increased in NOGDs (*t* = 2.16, *p*=0.040), and the FC between LFPN and DMN was significantly decreased in NOGDs compared with HCs (*t* = 2.1, *p*=0.045).

### 3.4. FNC Analysis

**Figure 3:**
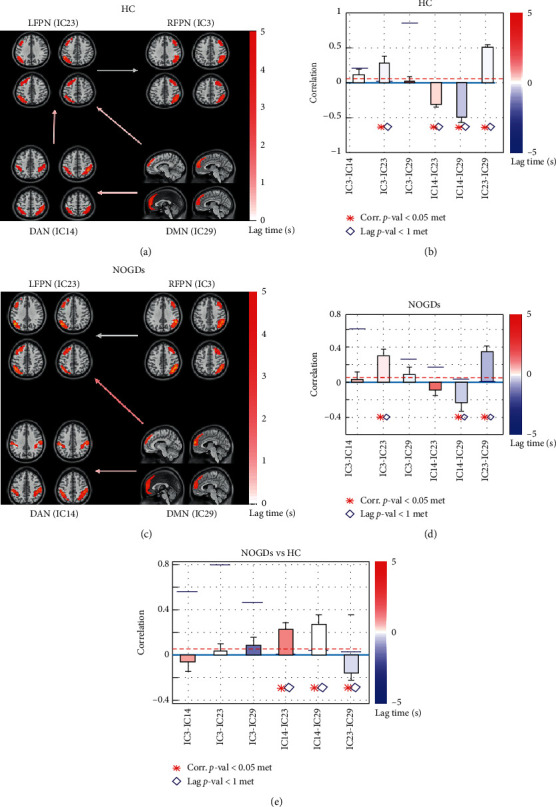
FNC analysis in the HCs and NOGD patients with SDS. *Note.* Corr, correlation; DAN, dorsal attention network; DMN, default mode network; FNC, functional network connectivity; HC, healthy control; IC, independent component; LFPN, left frontal-parietal control network.

The maximal lagged correlation coefficients of the ICs were calculated in the DAN, DMN, LFPN, and RFPN between NOGDs and HCs. The coefficients between the four components of two groups are displayed in Figure 3. The HCs showed a significant positive correlation between RFPN and LFPN and LFPN and DMN as well as negative correlation between DAN and DMN and DAN and LFPN. All of the significant correlation coefficients between the two components in NOGD patients were the same as the HCs except for the coefficient between DAN and LFPN. Two-sample *t*-tests showed the significant positive correlation coefficients between the DAN and LFPN and DAN and DMN as well as negative correlation between LFPN and DMN.

### 3.5. Correlation Analysis

**Figure 4:**
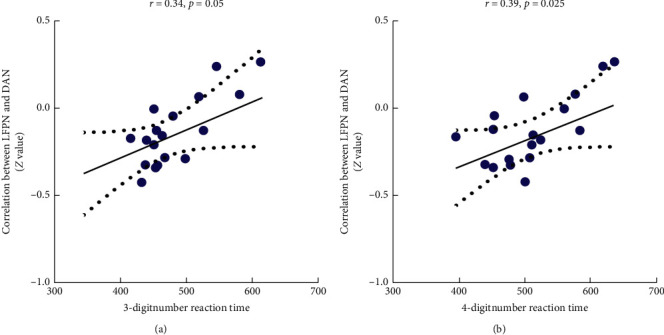
Alerted functional connectivity correlate to reaction time of CPT. *Note.* CPT, continuous performance test.

The regression analysis had been performed to explore the correlations between the FC values and scores of CPT (shown in Figure 4). The FC values between LFPN and DAN showed positive correlations with 4-digitnumber RTM (*r* = 0.39, *p*=0.025) and 3-digitnumber RTM (*r* = 0.34, *p*=0.05).

## 4. Discussion

Our main findings of this study were as follows: (1) the increased FC between DAN and LFPN and the decreased FC between LFPN and DMN in NOGD patients with SDS compared with HCs; (2) positive correlation coefficients between the DAN and LFPN and DAN and DMN as well as negative correlation between LFPN and DMN in NOGD patients with SDS compared with HCs; (3) positive correlations between FC values of network connectivity and CPT scores. Our preliminary findings suggested that relatively stable abnormal interactions occurred among the RFPN, DAN, LFPN, and DMN in NOGD patients with SDS.

In this study, CPT scores showed decreased scores of 2-FT, 2-RTM, and 4-RTM compared with HCs, which suggested that NOGD patients with SDS had attention deficits. Our result was in line with that of previous studies. One study revealed that patients with irritable bowel syndrome (IBS) showed lower attentional control in comparison with HCs [19]. Another study also on IBS patients showed specific abnormalities in attentional network functioning, which were correlated with symptom severity [20]. According to the Huangdi Neijing, spleen stores Yi and domain thoughts. Yi is thought to be related with multidimensional cognition including the attention [21, 22]. Hence, our results supplied evidence supporting the notion the function of spleen is associated with attention.

In previous studies, the DAN was confirmed to be involved in attention-oriented control, whereas the FPN played a vital role in goal-directed cognition [23]. The DMN exhibited decreased activation during cognitive tasks. The FPN-DMN and DAN-DMN both showed a pattern of anticorrelated relationship in both tasks requiring externally directed attention and resting-state studies [24]. Furthermore, numerous studies demonstrated that the FPN could regulate the dynamic balance between the DMN and DAN [23, 25]. One recent study revealed disrupted FC between the FPN and DMN in schizophrenia, which linked metacognitive deficits with clinical symptoms [26]. Another study focusing on the healthy subjects after sleep deprivation showed the decreased FC between FPN and DMN compared with the awake state, which was positively correlated with working memory task [27]. The study on mild cognitive impairment patients, young adults, and healthy elders also indicated that the anticorrelation between the DAN and DMN was associated with age, cognitive impairment, and disease duration [28]. These studies were in line with our FC and correlation results. Therefore, we postulated that the increased FC between DAN and LFPN and decreased FC between LFPN and DMN represented a functional compensation, which interpreted that NOGD patients with SDS tend to recruit more resources to support attention and executive control. Moreover, we performed the temporal correlations between the four subnetworks. The results also showed abnormal associations between LFPN and DAN and LFPN and DMN. One recent study had revealed that the FPN exhibited a higher positive correlation with DAN and more negative correlation with DMN during finger tapping task compared with during resting state [29]. Hence, our results strongly supported the dysfunctional interactions among LFPN, DAN, and DMN, which might be potential biomarkers for treating NOGDs.

However, some limitations of the current study should be pointed out. First, as a preliminary study, the number of recruited subjects in the current study was relatively low. The studies with a larger sample should be needed to strengthen our results in the future. Second, since the sample size is limited, we did not analyze different types of spleen deficiency syndrome. It would be better to conduct further analysis in future studies.

## 5. Conclusions

In summary, we found that the relatively stable changed relationships occurred among the DMN, RFPN, LFPN, and DAN in NOGDs with SDS, which were correlated with CPT scores. Our study might supply novel insights into the relationship among the three neurocognitive networks in NOGD patients with SDS to deepen our understanding of the neuropsychological mechanisms of SDS.

## Figures and Tables

**Table 1:** The demographic information of NOGD patients with SDS and healthy controls.

Items	NOGDs with SDS (*N* = 20)	Healthy controls (*N* = 18)	*χ* ^2^ (*t*/*z*)	*p*
Gender (male/female)	6/14^a^	5/13	0.014	0.906
Age (years)	25.50 ± 3.41^b^	27.28 ± 3.61	−1.56	0.127
Educational level (years)	18.45 ± 3.36^c^	16.28 ± 2.40	−1.95	0.051

*Note.* ^a^Results from the chi-square test of the comparison between two groups; ^b^results from the two-sample *t*-test of the comparison between two groups; and ^c^results from the nonparametric test of the comparison between two groups.

**Table 2:** Comparison on scores of CPT in NOGD patients with SDS and healthy controls.

Items	NOGDs with SDS	Healthy controls	*t*/*z*	*p*
2-FT	1.15 ± 1.27	0.28 ± 0.46	−2.19	0.029
2-ANR	29.10 ± 1.07	29.56 ± 0.78	−1.53	0.126
2-RTM	449.41 ± 62.51	485.68 ± 75.82	−1.29	0.198
2-SD	95.20 ± 33.76	98.58 ± 43.57	−0.18	0.860
3-FT	1.15 ± 1.14	0.72 ± 0.57	−0.21	0.834
3-ANR	27.60 ± 3.15	27.72 ± 2.52	−0.48	0.630
3-RTM	484.91 ± 51.80	426.72 ± 53.43	3.41^#^	0.002
3-SD	101.67 ± 23.21	106.49 ± 27.28	−0.79	0.428
4-FT	2.30 ± 1.75	1.56 ± 0.51	−1.51	0.130
4-ANR	24.55 ± 5.33	25.89 ± 2.47	−0.18	0.859
4-RTM	522.34 ± 70.95	497.93 ± 60.76	−2.22	0.026
4-SD	103.92 ± 30.56	107.49 ± 16.00	−0.32	0.748

*Note.* ANR, actual number of responses; DM, directed memory; FT, false trial; RTM, reaction time mean; SD, standard deviation; #, results from the two-sample *t*-test of the comparison between patients with kidney deficiency syndrome and healthy subjects.

## Data Availability

The data used to support the findings of this study are available from the corresponding author upon request.
